# An ecosystem framework for understanding and treating disease

**DOI:** 10.1093/emph/eoy032

**Published:** 2018-10-09

**Authors:** Michael E Hochberg

**Affiliations:** 1 Institut des Sciences de l’Evolution, Université de Montpellier, 34095 Montpellier, France; 2 Santa Fe Institute, Santa Fe, NM 87501, USA; 3 Institute for Advanced Study in Toulouse, 31015 Toulouse, France

**Keywords:** antibiotics, cancer, immune system, resistance, microbiota, pathogens

## Abstract

Pathogens and cancers are pervasive health risks in the human population. I argue that if we are to better understand disease and its treatment, then we need to take an ecological perspective of disease *itself*. I generalize and extend an emerging framework that views disease as an ecosystem and many of its components as interacting in a community. I develop the framework for biological etiological agents (BEAs) that multiply within humans—focusing on bacterial pathogens and cancers—but the framework could be extended to include other host and parasite species. I begin by describing why we need an ecosystem framework to understand disease, and the main components and interactions in bacterial and cancer disease ecosystems. Focus is then given to the BEA and how it may proceed through characteristic states, including emergence, growth, spread and regression. The framework is then applied to therapeutic interventions. Central to success is preventing BEA evasion, the best known being antibiotic resistance and chemotherapeutic resistance in cancers. With risks of evasion in mind, I propose six measures that either introduce new components into the disease ecosystem or manipulate existing ones. An ecosystem framework promises to enhance our understanding of disease, BEA and host (co)evolution, and how we can improve therapeutic outcomes.

## INTRODUCTION

Despite the pervasiveness of parasites and cancers across animal species and in humans in particular, our knowledge of the ecology and evolution of disease itself is surprisingly limited. When disease is studied in detail, it is often through descriptions of signs and symptoms, or the characterization of the biological etiological agent (BEA) and of diseased tissue ultrastructure. Other study—focusing on population-level interactions—usually abstracts disease into a single variable such as aggressiveness or pathogenicity. Although both approaches produce insights into BEA–host interactions, they oversimplify disease by omitting what may be its single most important driving force—ecology.

I advocate the perspective that when examined through an ecological lens, disease has a characteristic structure. Investigating this structure and variations on it is important not only for understanding ecology and evolution in a fundamental type of habitat—the organism—but also to develop approaches that lessen the burden of BEAs in animal husbandry, wildlife, on endangered and domesticated species, and in the human population—the latter being the focus of this review. The usual approach is to treat BEAs with drugs, but these can fail due to selection for resistance. The two most familiar types in humans are antibiotic resistance and chemotherapeutic resistance in cancers.

Early in the 20th century, Paul Ehrlich encouraged the search for the ‘Magic Bullet’—a single drug that targets and cures a disease with minimal residual toxicity for the patient. Ehrlich [1] also argued for the accepted approach of the day *frapper fort et frapper vite* (hit hard and without delay), based on the construct that drug dose must be commensurate with the severity of the disease and delaying drug delivery allows BEAs to multiply and cause irreparable damage and possibly death. Ehrlich and his contemporaries knew that some parasites could resist chemotherapy but were unaware of the underlying mechanisms. Despite clear arguments for why attempting to eradicate BEAs risks selecting for resistance [2, 3], *frapper vite et frapper fort* is still widely regarded as the surest way to cure disease.

The above two challenges—understanding disease and improving therapeutic outcomes—are interrelated because achieving the former will help address the latter, and because both natural, evolved processes that remediate disease and therapeutic interventions have ecology at their foundation. Here, I develop a framework for addressing these challenges. Focus is on humans because of the rich knowledge of some of their diseases and the growing importance of finding solutions to drug resistance and the improvement of therapeutic outcomes. In particular, I describe how disease can be understood as an ecosystem, the components and processes of which can be either manipulated separately or in combination toward a therapeutic objective. The *disease ecosystem* employs analogies from terrestrial, aquatic and agricultural ecosystems (hereafter ‘classic’ ecosystems), including predators, prey, competitors, detrivores, resources and the environmental surroundings. Thus, like classic ecosystems, disease ecosystems are intrinsically ecological: they are influenced by the environment and composed of feeding relationships (food webs) among ‘species’ in an interactive community.

I begin by justifying the need for an ecosystem framework of disease. I argue that in both healthy and diseased tissues there is a largely undiscovered world of ecosystem-like processes including resource fluxes, inter- and intraspecific competition, predation by immune systems and waste removal. I develop this concept as the central feature of the framework and then focus on how the state of the BEA population and associated disease provide reliable information about disease progression or remediation. Before applying the framework to therapies, I discuss what failures due to resistance typically resemble and present several other routes to therapeutic evasion that have received little attention. Key to applying the framework is how decisions are made about whether a treatment should attempt to eradicate or contain the BEA, or rather simply limit disease, and how strategies and tactics can be either used singly or in combination. Finally, I present some limitations to the framework and concluding thoughts.

The framework is centered on BEAs that replicate in the disease ecosystem such as microparasites and cancers, and not asymptomatic infections or diseases stemming from non-BEAs. The current framework should therefore be viewed as part of a larger picture. I also do not address the important question of how to measure ecological and evolutionary features of disease (see reviews in e.g. [4, 5]). Finally, I do not attempt a systematic overview of the huge diversity of human BEAs. Rather, I refer where appropriate to specific examples in several well-studied BEA groups, principally bacteria and cancers. Comparing and contrasting the basic features of these groups is the first step towards an understanding of disease ecosystems and how they can be used to devise successful therapies.

## THE NEED FOR A GENERAL FRAMEWORK

Disease is a complex system of multiple species interacting at multiple biological and spatial scales. Whether and how a disease progresses, what are the implications for the BEA and the host, and how to design therapies can only be answered if we characterize the underlying components, their functions and interrelationships.

The limited accessibility and microscopic nature of disease makes it particularly challenging to study and to measure. As a consequence, *in vivo* work typically takes physiological and epidemiological approaches, describing signs and symptoms, changes in tissue ultrastructure, and consequences for morbidity and mortality. In contrast, *in vitro* study usually focuses on the BEA itself and abstracts-out the complexity characteristic of *in vivo* environments. This is problematic, for example, because *in vitro* observations can be misleading about *in vivo* behaviors [6]. The combined situation—the general ignorance of ecological and evolutionary mechanism *in vivo*, the oversimplification of real environments *in vitro* —limits our knowledge of how disease-generating processes actually work.

Beyond the importance of how both ecology and evolution influence disease, a general framework needs to have the flexibility to accommodate the effects of therapeutic interventions. Therapy can be viewed as an ecological perturbation intended to reset a disrupted system to its healthy state. As indicated above, the prevailing approach is *frapper fort et frapper vite* to eliminate BEAs and minimize the chances that (contagious) BEAs are transmitted to other hosts. However, a large body of work indicates that targeted antimicrobial and anticancer chemotherapies select for resistance [3, 7], and there is increasing awareness that drug therapies can have enduring disruptive effects on the microbiome [8] (but see [9]) and the host itself (e.g. contributing to other diseases, such as secondary cancers).

Despite the large body of evidence that treat-to-cure chemotherapy can lead to resistance and treatment failure, this approach has stood the test of time, largely due to its simplicity and common sense, but also because it often works [10]. Aggressive chemotherapy also continues largely unchallenged because scientific study is still in early days of evaluating alternatives, most of which are centered on preventing or limiting the evolution of resistance. Until relatively recently much of what was known about resistance and its management derived from pesticide use (Box 1). Although the lessons learned are foundational, they often abstract-out ecology, and are sometimes difficult to apply to disease due to differences in biology and ecology, habitat structure and habitat resilience to treatment.

**Box 1: The lessons of pesticide use**

Much of our knowledge about therapeutic resistance and how to manage it can be traced to lessons from the use of pesticides in agriculture [181, 182]. Pesticide treatments attempt to reduce damage below an economic threshold while not exceeding acceptable levels of residual toxicity for consumers and the environment (e.g. non-target species). By the very nature of pest status (i.e. large, dispersive populations with high potential growth rates) and high but nevertheless limited doses of chemicals (meaning that sufficiently resistant strains are likely to survive a given dose), the repeated, blanket application of the same ‘magic bullet’ over a population will eventually select for resistance. The usual course of action is to search for new compounds to replace the failing ones. But our ability to create new active substances is declining and the number of resistant pests is increasing [183]. Ecologically- and evolutionarily informed approaches have become promising alternatives. Primary among them is the prediction that resistance can only be contained by ‘conserving’ sensitive strains of the pest. For example, the evolution of resistance can be slowed if the competitive balance is shifted in some places and times in favor of sensitive strains [184]. There is evidence that this and other tactics increase the likelihood of managing resistance in pest control programs [185].

We therefore need a general framework that is firmly rooted in ecology and evolution both as a basis for fundamental understanding and to guide therapies [11]. There are a number of developments and frameworks that integrate ecology and evolution (parasites and pathogens: [12–15]; cancers: [5, 16–20]), but none extend to both infectious and non-infectious BEAs. My aims are to generalize the disease ecosystem concept and to use its insights to suggest several novel therapeutic interventions.

## AN ECOSYSTEM FRAMEWORK OF DISEASE

**Figure 1.**
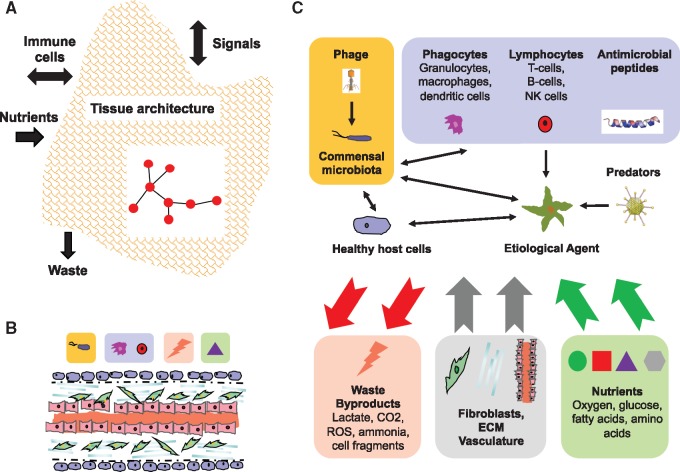
Healthy and disease ecosystems. (**A**) Organ- and tissue-level scale of the main compartments in host ecosystems. The boundaries of the ecosystem are determined by the interactions and events of importance and interest to the observer. Organ type, tissue architecture and local environmental conditions will play roles in the structure and dynamics of the ecosystem. In particular, interactions among cells (depicted as a network) and immune system flows into and out of the reference ecosystem will mediate homeostasis and/or dysbiosis in association with the BEA. (**B**) Basic structure of a healthy tissue ecosystem. This consists of the local vascular system, epithelial cells, extracellular matrix (ECM), microbiota (e.g. bacteriophages and bacteria) and elements of the immune response, including phagocytes, lymphocytes and antimicrobial peptides. Nutrients delivered through the vascular system feed nearby living cells. Foreign cells may be engulfed by phagocytes, and waste removed by both phagocytes and the vascular system (diffusible wastes, e.g. CO_2_). Finally, fibroblasts as part of the innate immune system contribute to maintaining tissue structure (ECM and vascular system) and initiating the immune response to injury or BEA invasion. See caption C for key to symbols. (**C**) Basic structure of the disease ecosystem. BEAs, microbiota, their natural enemies (e.g. viruses) and immune cells interact in a community. Healthy host cells are also part of the community since they compete locally with BEAs. All living cells in this disease ecosystem consume nutrients and produce waste and by-products, some of the latter two of which is recycled. Like the healthy tissue ecosystem, the habitat is supported by fibroblasts, ECM and the vascular system, but, notably in the case of tumor microenvironments, the structure of these are disrupted by the damage caused by the BEA and chronic inflammation (not shown). Arrows indicate a subset of the possible directions of influence. See main text for details

**Figure 2.**
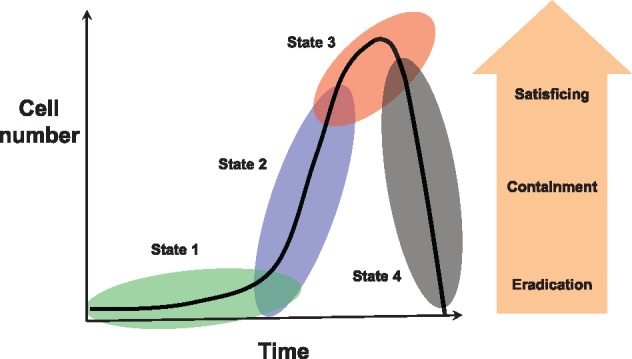
Four states in BEA and associated disease. This classification is based on growth in a novel and hostile environment (State 1), BEA population size (cumulative number of births) and therefore evolutionary potential (State 2), and spread and colonization of local and distant within-host habitats with correspondingly greater impact of disease on the host (State 3). Therapeutic objectives (eradication, containment, satisficing) change with progression through the three first states (it is assumed that natural remission (State 4) will not be subject to therapeutic intervention). In particular, although optimal protocols are feasible in States 1 and 2, treatment options may be limited to ‘satisficing’ in (late) State 3, for example due to reduced tolerance to drug toxicity. Should the immune response and any corresponding inflammatory response be sufficient, then the BEA and associated disease would regress to the healthy tissue state and homeostasis (State 4). This is shown for remediation from State 3, but it could also occur at States 2 or 1. See main text for further discussion

The framework for understanding and treating disease (Figs 1 and 2) focuses on how the components and processes in the disease ecosystem interact directly and indirectly with the BEA. The system can be in one of four main states that reflect BEA emergence, population growth, spread and pathogenicity, and regression. Understanding the state of the BEA and associated disease will be the basis for applying the framework to therapeutic interventions, as described below.

### The organism ecosystem

The ecosystem concept is a general way of representing species interaction networks, non-living organic and inorganic matter pools, and the physical habitat. Key to the concept is the fluxes, balances and stocks in these different compartments, and how events (e.g. environmental disturbance, species invasions) can have consequences for individual compartments or the ecosystem as a whole.

Similar to classic ecosystems, the healthy organism ecosystem is composed of supportive and regulatory structures (Fig. 1A and B). Supportive structures include habitats (tissues), resource replenishment (circulatory system) and waste removal (phagocytosis, circulatory system). Regulatory structures include cooperative interactions (cell–cell signaling, hormonal control), predation (immune systems), and commensalism, competition and parasitism (microbiota). These structures will vary between tissue types [14], suggesting that ecosystem dynamics may contrast as well.

Although it is tempting to perfectly equate different components in organism ecosystems with analogs in classic ecosystems, they do have at least one important difference: whereas Darwinian selection acts on many component species in the latter, it is centered on a single species in the former (but see [21]). Thus, we would expect the core of the organism ecosystem (i.e. host cells) to evolve traits that promote habitat maintenance and remediation, meaning more coordination and less autonomy than species interactions in classic ecosystems. We would nevertheless also expect that similar processes are at work between the two system types, including selection acting on traits ultimately affecting the ecosystem, and co-evolution with mutualists, commensals and parasites. More research is needed to explore these observations and expectations.

### The disease ecosystem

The disease ecosystem results when a healthy ecosystem is disrupted by a BEA and the host organism attempts remediation. This is in some ways analogous to environmental disturbances in classic ecosystems and more specifically to those stemming from invasive species (Box 2). Below I describe some of the main features of the disease ecosystem (Fig. 1C), and briefly review the most understood type—the ‘tumor microenvironment’—in Box 3.

**Box 2: BEAs and invasive species**

BEAs have commonalities with invasive species [186]. They colonize a spatially heterogeneous and potentially hostile environment, and may adapt to or transform the prevailing host ecosystem into one where they can grow, evade predation (or grow despite it), gain access to resources, and multiply and disperse. In so doing BEAs interact with the microbiome, host cells and tissues, and produce waste. Moreover, like invasive species, BEAs can be spatially structured. For example, Lloyd *et al.* [187] examined spatial models of tumor growth and found that cells toward the center of the tumor aggressively competed for resources and attained high densities, whereas cells toward the periphery grew faster and competed less. Similar to invasive species [188], BEAs may be associated with specific microbial communities (e.g. [14, 140, 189, 190]), but any causation between the two can be difficult to determine (see [191]). Also, BEA activity may lead to the damage of host cells, tissues and organ systems, and the impairment of waste removal, and there are parallels with invasive species in classic ecosystems [192].

**Box 3: The tumor microenvironment**

The most understood type of disease ecosystem is the ‘tumor microenvironment’. One principal difference between tumor cells and other BEAs is that the former are - themselves - diseased. Tumor cells divide, move, compete and may cooperate in a 3D semi-structured mass [193–196]. However, only those tumors successfully evading the immune response will progress to cause significant disease [65, 85]. Similar to healthy host cells or growing parasites, tumor cells sequester resources from the host. But in contrast to the first two, tumor cells dramatically increase their glucose uptake through aerobic glycolysis and produce waste in the form of lactate (i.e. the Warburg Effect [197]).

Spatial structure plays an important role in the disease ecosystem of solid tumors. Because of the disruption of otherwise healthy tissue, diffusible wastes increase and nutrients decrease with distance to the nearest capillaries. This is particularly important in cancer growth, where cells toward the interior of a tumor tend to be deprived of resources (glucose and oxygen) and exposed to high concentrations of various wastes (especially lactate). These conditions can lead to altered cellular behavior or cell death [198], and can even extend beyond stressed areas of a tumor: hypoxic cells may diffuse molecular signals that stimulate (i) angiogenesis thereby fostering cell survival and tumor expansion, and (ii) cell motility, which promotes metastasis [199].

Another characteristic of the cancer disease ecosystem is that massive tumor cell division and genomic instability of tumor cells generate considerable phenotypic variability (State 2, Fig. 2), some of which will increase adaptation to changing micro-environmental conditions. Adaptive traits include changes in cellular growth (r or K) strategies, escape from immune responses, movement away from inhospitable microsites and dispersal to other tissue ecosystems in the body [34, 194, 200–202]. Although the details differ, many of the above elements are analogous to interactions in classic ecosystems and in food webs [203].

When the BEA enters or emerges from within a healthy host ecosystem it is likely to be confronted by one or more of physical structures [22, 23], oxidative stress, chemical defenses [24] and innate immune responses [25]. Immune systems in particular are central to somatic maintenance, including the host’s ability to either limit the growth and spread of (or eradicate) a BEA [26], and to repair tissue damage [27]. However, some BEAs may evade the immune response, grow, spread and generate disease. BEA and host genotypes, the environment and tissue type are among the many factors influencing these processes and the associated severity and extent of disease [25, 28–31].

Many disease ecosystem interactions involve vying for resources. As the BEA grows it competes for space and/or nutrients and this may result in stressful local conditions (e.g. hypoxia, [32, 33]), and in certain cancers, favor disease progression [34]. Competition for space or nutrients can occur with the same or other BEA types [35–37], host cells [38] or commensal microbiota [39]. For example, Balmer and Tanner [37] argued that multi-strain infections could have far reaching consequences for the ecological and evolutionary dynamics of disease, including mutualistic interactions and direct and indirect competitive interactions (e.g. apparent competition). Nutrient levels have also been shown to influence BEAs indirectly via the immune system [40]. Despite the multitude of potential indirect interactions suggested by Fig. 1C—particularly with the microbiota—little study to date has investigated these in detail (e.g. [41]).

Although many of the events in disease ecosystems have analogs in classic ecosystems, the detailed functions often contrast. Thus, for example, whereas immune systems and predators ultimately kill their ‘prey’, unlike predators, immune cells do not appear to gain significant energy in the process. Rather, the latter derive energy from nutrients *in situ*, including in the disease ecosystem (for the tumor microenvironment, see [42]). Moreover, unlike predators, different immune cells cooperate in killing diseased/damaged cells and pathogens. (A notable exception to this in classic communities is cooperative hunting.) Thus, lymphocytes function as precursors for phagocyte activity, either marking or killing target cells, whereas phagocytes engulf and remove target cells and waste. Despite detailed knowledge about immune systems, we know very little about their functional and numerical responses (but see [43]) and how these may contrast with classic species communities.

### States of BEA and associated disease

The BEA is at the center of the disease ecosystem framework. The BEA population is dynamic, yet proceeds through one or more of a characteristic series of states. These states can convey considerable information about the current and future growth of the BEA and disease. Torres *et al.* [44] proposed a ‘disease map’ that tracks pathogen load and patient health through the course of a disease. Below, I reinterpret this concept emphasizing the growth and evolvability of the BEA population and associated disease (Fig. 2).

#### State 1. Establishment: small numbers, low evolutionary potential, negligible disease

As described above, during establishment, BEAs confront novel and possibly hostile environments [45, 46]. Establishment will depend on the BEA’s ability to evade immune responses and either already possess or plastically express virulence factors permitting resource acquisition and growth [47]. Should the immune response be sufficient, the BEA enters directly into State 4 (see below) and declines. Even should a small BEA population continue to grow, its total mass and the extent of associated disease is likely to be minute. State 1 continues until (i) cumulative cell turnover is on the order of the reciprocal of the beneficial mutation rate (entry into State 2), and/or (ii) the intensification of disease due to some combination of direct damage by the BEA and possible chronic inflammation (entry into State 3).

Detailed study of the initial phases of BEA population growth is uncommon. For example, Margolis and Levin [48] considered how commensal *Haemophilus influenzae* could become invasive from the nasal passage to the blood and then evolve. They showed that invasive infections have a significant stochastic component, are typically initiated by a single bacterial cell and found evidence for infrequent within-host evolution. Leggett *et al.* [49] looking across 43 different human pathogens found that lower inoculum numbers were required for infections generating pathogenesis at local as opposed to distant sites. The former tended to be more virulent than the latter. In contrast to parasitic BEAs, tumorigenesis requires key initiating mutations that compromise cooperative cellular functions [50], and together with subsequent tumor progression and metastasis (States 2 and 3), this can take years or even decades to achieve (e.g. [51]).

#### State 2. Growth: mutation, adaptation and resistance

The transition from a relatively slow growing, evolutionarily limited BEA in State 1 to an exponentially growing, evolving BEA in State 2 is associated with both population turnover (total births per unit time) and mutation rate [52, 53]. Population growth will be influenced by the host immune system and nutrient levels [40, 54]. The per-birth mutation rate can be highly variable between different BEA strains and types, with more rapid passage from State 1 to State 2 expected for hypermutating bacteria, RNA viruses such as HIV-1, and genomically unstable tumor cells. For example, Sottoriva *et al.* [55] studied the spatio-temporal dynamics of beneficial mutations in colorectal cancers. They found that the first dominant mutations emerged in tumors containing as few as *∼*<10 000 cells, which can be explained in part by mutation rates as high as *∼*10^−5^ (see also [56]).

BEA population growth and evolutionary potential have two notable consequences. First, growing BEA populations result in alteration of host cell behaviors and for some BEA types, greater tissue damage. Second, higher mutation rate favors anti-immune defenses [57], adaptations in nutrient use [58, 59] and resistance to therapeutic interventions [60, 61].

#### State 3. Spread: growth and dispersal magnify disease

Continued BEA growth eventually results in metabolic competition and—particularly in the tumor microenvironment—waste accumulation. One or both of these factors can promote local colonization and migratory behaviors [62, 63], which lead to increased pathogenicity. Although disease can be significant at low BEA densities (States 1 and 2), all else being equal, it usually worsens with time and as BEA numbers grow (see contrasting scenarios in [64]). Cancers are arguably the best examples of how growth, local tissue invasion and distant tissue colonization—together with chronic inflammation—compromise host condition and increase mortality. Local tissue invasion and metastatic spread are indicative of escape from immune control [65]. Similar patterns are observed in certain microparasites, where disease progression is associated with local tissue invasion and cell entry [23].

#### State 4: Regression: immune responses abate BEA and disease

Should the combined effects of the innate and adaptive immune systems prevail resulting in BEA population decline, then disease too will eventually regress [44]. Depending on immune and BEA dynamics, State 4 may obtain at any point during States 1 to 3 for infectious BEAs, whereas for cancers State 4 is most likely to obtain during State 1, before the tumor evolves to evade the immune response. BEAs in State 3 that do not enter State 4 either lead to chronic disease or result in host death.

The above classification gives a clear message for therapy. A disease is much more treatable in State 1, but either impossible or difficult to diagnose compared to State 2 and particularly State 3. In States 2 and 3, resistance is a concern, and special measures may be needed to treat the BEA. Finally, drug toxicity becomes an issue as the BEA population grows, disease spreads, and the person’s health is at risk or life is engaged, as would be observed (late) in State 3. Observation that the BEA and associated disease are in State 4 would often preclude a therapeutic intervention.

## APPLYING THE FRAMEWORK

The population ecology of BEAs makes them particularly difficult to eradicate. Once disease is detected, BEAs typically will have attained large population sizes and generated mutant strains that result in opportunities for evolutionary responses. There are useful parallels between seeking to cure a disease and attempting to eradicate an invasive species. Perhaps, the most compelling is that eradication becomes more achievable as the population is smaller and more spatially circumscribed (States 1 and 2, Fig. 2). Thus, for example, invasive species are more difficult to eradicate on mainland (or interconnected habitats) than on islands (or isolated habitats) [66]. Both ecological dynamics (e.g. larger population sizes, refugia) and evolutionary dynamics (e.g. greater additive genetic variation) contribute to explaining this observation. The same basic principle applies to treating a BEA, where diversification (State 2) and spread (State 3) make eradication less probable, as is amply demonstrated by relapse due to chemotherapeutic resistance in metastatic cancers.

Below, I first review different ways in which therapies fail. I then present six eco-evolutionary-sensible measures that can be used singly or in combination to achieve therapeutic objectives.

### Why do therapies fail?

Excepting technical issues of inappropriate drugs, the main probable cause for treatment failure is BEA resistance [10, 67]. Drug resistance due to high-dose therapy is a textbook example of ‘evolutionary rescue’ [68, 69], where the drug (if it were to be maintained) drives the sensitive population to extinction, but resistant variants already present or emerging during the treatment grow and repopulate the disease ecosystem. The actual sequence of mutational events leading to rescue has rarely been studied [70], and we are only beginning to learn about the underlying ecological processes (see [69] for contrasts between medicine, agriculture and conservation biology). For instance, although the evolution of resistance is often associated with high-dose chemotherapies, lower dosing (e.g. to regulate toxicity, especially in cancer chemotherapies) can also select for resistance [71, 72].

The most discussed mechanism leading to evolutionary rescue is ‘competitive release’, whereby reductions in the population of the sensitive strain open the niche for growth of otherwise competitively suppressed resistant strains [73, 74]. Qualifying all instances of evolutionary rescue as stemming from competitive release is an oversimplification, because there are notable contrasts in the mechanism depending on the absolute and relative fitnesses of sensitive and resistant strains, both in the presence and absence of a drug [74]. Nevertheless, there is some support for the general phenomenon of competitive release [75–77].

Much of what is known about treatment failure comes from studies of the evolution of drug resistance. But barring data actually demonstrating resistance, other less studied, non-mutually exclusive mechanisms may be involved in BEA resurgence or relapse.

*Competitive release*—although sometimes used for inter-strain competitive effects (see above)—is originally an ecological concept [78]. In ecological competitive release, the elimination of the target BEA results in the emergence of one or more otherwise competitively suppressed BEAs [79].

*Therapeutic tolerance* is the ability of BEAs to withstand the transient effects of a treatment. Examples include reduced receptor sensitivity or density-limited drug impact in cell sub-populations of certain cancers [80], and quiescent or dormant states in cancer stem cells [81] and bacterial persister cells [82]. Other forms of tolerance involve protective structures, such as bacterial biofilms against antibiotics [83], biofilms limiting host immune responses [84], and immune tolerance in the tumor microenvironment [85].

*Escape mutants* do not have specific resistance or tolerance mechanisms, but rather simply outgrow the suppressive effects of the therapy. This may occur, for example, due to insufficient drug dosing [86, 87].

*Spatial and temporal refuges* are similar to escape mutants, except that BEA survival is due to heterogeneity in therapeutic exposure. Examples include limited drug diffusion [88–90] and ‘drug holidays’ or patient non-compliance [86].

*High mutation rates* may favor escape by increasing the chances of resistance emerging in bacteria [91], HIV [92] and certain cancers [93]. Hypoxic stress in leading to higher mutation rates in tumors could explain certain origins of therapeutic resistance [33].

### Therapeutic measures

Applying the framework involves first acquiring information about the BEA and disease ecosystem (Figs 1 and 2). A therapeutic objective is chosen based on this information and on actionable strategies and associated tactics. The choice considers failure risks due to BEA resistance or evasion. Typically, a single strategy and tactic will be deployed to achieve an objective, but as will be explained below, under some circumstances different strategies/tactics may complement one another and become a combination strategy. Below, I refer to strategies and tactics as ‘measures’. A treatment is deemed a success if it achieves its objective (see Supplementary Material for a simple criterion).

Importantly, a chosen measure need not do all the ‘work’ to achieve a therapeutic objective. Rather, it can be employed as an adjuvant either to other measures as part of a combination strategy or to processes already active in the disease ecosystem. For example, amoxicillin is a common antibiotic used for treating type A strep throat associated with *Streptococcal pharyngitis*. The antibiotic interferes with BEA cell wall synthesis and thus targets bacterial replication. Amoxicillin may not be 100% effective on its own (due to limited pharmacokinetics) and different components of the immune system ensure the final stages of the complete clearing of the infection [94].

**Figure 3.**
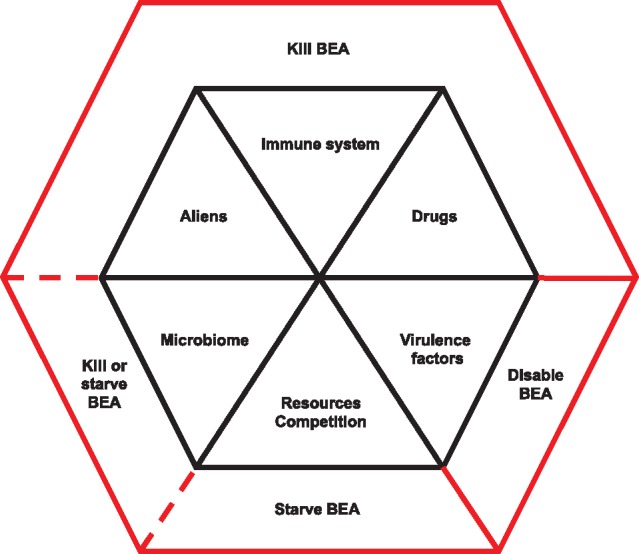
Six measures for treating disease. Measures have one or more of three proximate effects: kill (cytotoxic), disable (cytostatic) or starve the BEA. Killing the BEA is the most likely of the three to select for resistance. Combination therapies (two or more measures) are most likely to succeed if they do not interfere with one another in mode of action and do not select for resistance in the same or in linked genes. See main text for further discussion

Below, I present a series of six measures for treating disease (Fig. 3), starting with the most employed—drug-based targeted therapy (Measure 1). Measure 1 has three main variants depending on the objective: eradicate, contain or satisfice. A second approach is to focus on reducing damage, rather than target the BEA *per se* (Measure 2). A third approach is to target other components of the disease ecosystem (Measures 3–6). Finally, I describe the strategy of combining measures into more effective therapies.

#### Measure 1: Drugs

Alexander Fleming expressed a nuanced version of *frapper fort et frapper vite* [95]. His recommendation ‘if you use penicillin, use enough’ was based on the idea that sensitive strains were the origin of *de novo* resistant mutants, and the former needed to be eliminated quickly and decisively. Resistant mutants may however be present even before a therapy commences, either in the pathogen inoculum (or the first cells of an invasive carcinoma) or due to mutations in a growing BEA population (i.e. State 2, Fig. 2). The observation that the Ehrlich–Fleming approach applied to a large enough BEA population risks selecting for resistance has fueled theoretical research aimed at determining the drug doses and scheduling that will either contain or eradicate a BEA (e.g. [96–101]). These studies and others have contributed the following basic insights.

##### Eradicate

If the turnover of the BEA population and/or its genetic diversity are low (State 1), then resistance mutants are unlikely to be present [53] or may fail to emerge even under treatment (see also [102]). Eradication becomes an option. Depending on the information available about possible resistance mutants, the minimal dosing strategy is (in order of decreasing dose) either to apply the maximum tolerated dose (no information), attain the mutant prevention concentration [103] (information about resistance) or apply the dose necessary to clear sensitive strains (no resistance detected).

##### Contain

When the turnover of the BEA population and/or genetic diversity are high (States 2 and 3), then high-dose, targeted therapy is likely to be useless or even counterproductive. Examples where this may occur include clonal expansions [16], and genetically diverse infections [104]. Often the course of action taken when resistance is suspected or identified is to employ particularly aggressive drugs or—for some multi-drug-resistant pathogens—last resort drugs [105]. These treatment strategies, and indeed intermediate dose treatment strategies, are likely to fail [101, 106, 107]. Experimental and theoretical study indicates that the most sensible approach is to aim to contain the BEA through low but sufficient dose chemotherapies (e.g. [101, 108], and references therein). Containment requires that sensitive BEA strains be held in check by the therapy, but maintained at levels so as to competitively limit the populations of any drug-resistant strains when therapy is released. This is most readily achieved through managing the total BEA burden, whereby resistant types express a fitness cost relative to sensitives during periods of relaxed therapeutic selection (recent reviews in e.g. [98, 109]). More nuanced ‘adaptive’ approaches monitor therapeutic performance and eventually adjust drug dose or drug type so as to manage disease burden [110]. Recent experimental demonstrations of tumor containment show some promise (*in vitro* [111]; *in vivo* [77]), and the same approach should be applicable to certain microbial pathogens [112]. There are nevertheless situations where containment strategies are unlikely to succeed, such as when fitness-compensatory mutations have emerged in resistant strains during evolutionary rescue or have been transferred horizontally (e.g. [113–115]).

##### Satisfice

Simple dosing rules can be misleading when toxicity is an issue (late State 3 and no indication of entering State 4), as is often encountered in metastatic cancers. This is because the doses needed to optimally treat the (advanced) BEA are too toxic for the weakened patient. This situation becomes particularly acute when the patient’s life is engaged and rapid decisions need to be made [116]. In such scenarios aiming for ‘optimality’ is better replaced by ‘satisficing’, where success is equated with an acceptable improvement in signs and/or symptoms [117]. Much of what is known about containing a BEA while managing toxicity through scheduled dosing comes from theoretical study. For example, Foo *et al.* [118] examined pharmacokinetic models of the emergence of drug resistance in patients with epidermal growth factor receptor lung cancers. They found that the pulsed dosing (to control toxicity) could limit tumor growth, but long intervals risked the emergence of new drug-resistant clones.

Drug therapies are not ‘stand-alone’—particularly when targeting a microparasitic infection. Unless a patient is immunosuppressed, the immune system will act in conjunction with the treatment. This means that optimal dosing may depend on host condition (drug tolerance, ability to mount an immune response) and the disease ecosystem (the BEA population state). For example, Ankomah and Levin [119] studied a model of how the innate and adaptive immune systems could complement the effects of a drug to clear a bacterial infection. They showed that high drug doses led to the most successful outcomes (but see contrasting findings in [101]).

Despite empirical work showing that drug dose can influence treatment outcomes (for literature survey, see [101]), the necessary information required to make strategic decisions is often lacking. This could in part explain why eco-evolutionary reasoning is not being used to its full potential in deciding whether and how to employ aggressive treatment or containment strategies [98, 104, 107].

#### Measure 2: Virulence factors

The severity of disease will be influenced by the expression of ‘virulence factors’ that enable the BEA to construct its niche. Virulence factors may promote resource extraction, protect from host defenses, or attach to cell or tissue surfaces. They are most documented for microparasitic BEAs and include adhesive structures, motility enhancers such as Type IV pili, and toxins [120, 121]). Virulence may also have a social component, for example via quorum sensing in certain bacteria [122, 123].

Anti-virulence drugs have gained attention as possibly being ‘evolution proof’, because the traits actually targeted have little influence on fitness [120, 124]. Examples of drug targets include rendering bacteria more susceptible to immune clearance and increasing antibiotic efficacy [125], and cytostatic drugs that stall cancer cell division rather than targeting cell survival [126]. Other approaches seek to change BEA behaviors, such as increasing pH in cancer microenvironments that reduces tumor growth and metastasis [127]. Although *in vitro* studies of anti-virulence strategies appear promising (e.g. [128]), resistance is a concern, and there may be limitations in their efficacy *in vivo* [117, 120, 121].

#### Measure 3: Immune system

The immune system is the single most important mechanism responsible for preventing disease and limiting its spread. Immune systems can be remarkably complex, and their detailed description is beyond the scope of this review. Broadly speaking, immune responses may involve specialized molecules and/or cell types. Introducing molecules from the innate immune response—notably antimicrobial peptides—shows great therapeutic potential by both their stunning diversity and the lower likelihood of resistance evolution compared to antibiotics [129]. Immunotherapies that stimulate the production of specific immune cell motifs show promise in treating certain cancers [130] and bacterial diseases [131], but their use is still controversial due to risks of immunotoxicity and autoimmune disorders [132]. Related to this, Smyth *et al.* [133] recently argued for using information about components of the tumor microenvironment in decisions about immunotherapies, particularly as adjuvants to other therapies.

#### Measure 4: Resources—competition

BEAs need resources to grow and multiply. Resources include hospitable space and nutrients such as glucose, oxygen, carbon, nitrogen and phosphorus. Glucose use in particular differs fundamentally between healthy cells and tumor cells (with the latter consuming up to 18 times the former; [134]), as does phosphorus [135], and leveraging these may contribute to a strategy for tumor control. Resource deprivation by curtailing angiogenesis has proved successful in certain cancer treatments, but can come at a cost of limiting drug diffusion through the tumor [136]. In *Plasmodium,* Wale *et al.* [137] recently showed how resource limitation could differentially impact sensitive and resistant strains. Other tactics either limiting or excluding BEAs include boosting the competitiveness of healthy cells [138], or of commensals in the microbiome [139].

#### Measure 5: Microbiome

The microbiome is increasingly recognized as foundational to organism biology and condition. Some analogies are likely to apply between the functions of microbes in classic ecosystems and in healthy and diseased ecosystems [15]. For example, dysbiosis may be associated with certain BEA infections [121] and cancers [140], and there is accumulating evidence that the gut microbiota modulates the effectiveness of cancer therapies and associated toxic effects [141].

That microbiome disruption can influence disease suggests that re-normalizing it could prevent or ameliorate certain diseases. For example, lifestyle changes (e.g. avoiding high caloric intake and excessive hygiene) may indirectly prevent disease via their impacts on the microbiome, and more active interventions such as microbiome transplants could either be used preventively or therapeutically [140, 141].

#### Measure 6: Aliens

‘Living drugs’ such as bacteriophages (or ‘phages’) are akin to introducing a predator to control an agricultural pest. Phages self-amplify and adapt to their bacterial hosts, meaning that they can continually counter bacterial resistance [142]. In addition to their use as self-propagating, evolving antimicrobials, bacteriophage can be applied prophylactically to prevent bacterial pathogens from emerging [143], or engineered and introduced to disrupt antibiotic resistance [144]. Although less studied, replicating oncolytic viruses have clinical potential similar to baculoviruses [145], but with the added twist that under some circumstances they can induce anti-tumor immunity [146].

Similar to drug therapies, when submitted to phage predation, sufficiently large and diverse bacterial populations evolve resistance. Although a phage population will typically respond by evolving countermeasures, a more proactive, promising strategy is to evolutionarily ‘train’ phage before they are actually employed [147, 148]. Training involves selecting phage *in vitro* for traits that impact current bacteria at the infection site and prevent the future emergence of resistant strains. *In vitro* study indicates that passaging phage achieves the former objective, whereas coevolving them with the bacterium achieves the latter [148–150]. Training oncolytic viruses on tumor cells *in vitro* remains unexplored.

### Combining measures

An important principle from integrated pest management is that attaining a control objective is more likely as multiple, complementary practices are put into place. This same idea—combining two or more variations of the same measure or two or more measures—is therapeutically sensible both ecologically and evolutionarily [151]. Combination therapies are most likely to succeed when each measure (i) reduces BEA numbers or turnover, thereby removing existing resistance mutations and lowering the probability of such mutations emerging *de novo* to other measures (e.g. therapy A kills single mutants with resistance to therapy B, and vice versa) and (ii) acts through a different, non-antagonistic mechanism in targeting the BEA. Both of these effects imply that different genes are involved in resisting different measures. Crucially, because in sufficiently small populations it is unlikely that multiple resistance factors will occur in the same BEA individual, combinations are less likely than single measures to select for resistance [86, 152, 153] (see [60, 154] for possible exceptions in tumors).

Combination therapies have become a mainstay for treating HIV, malaria and tuberculosis and are particularly relevant to cancers [133, 145, 155]. They are also effective against bacterial pathogens, for example, the use of phage cocktails [156, 157], multiple antibiotics [158, 159], certain aminoglycosides and metabolites against persister bacteria [160], antimicrobial peptide cocktails [161], phage–antibiotic combinations [157], and phage to select for increased sensitivity to antibiotics [162]. Like phage–antibiotic combinations, antibiotic combinations that target different critical bacterial functions may not only increase the chances of therapeutic success but also limit the evolution and spread of antibiotic resistance [163]. Due in part to their great diversity, combinations involving natural host-derived antimicrobial peptides are particularly promising in this regard [164].

Depending on the nature of their interactions, measures can be combined either simultaneously or sequentially. For instance, Roemhild *et al.* [165] demonstrated how the order of sub-lethal doses of antibiotics could have substantial effects on bacterial population sizes and resistance (see also [96]). Torres-Barceló *et al.* [166] showed how intermediate delays between phage and antibiotic application not only minimized numbers of *Pseudomonas aeruginosa* but also minimized resistance to both antibiotics and phage. Sequential applications have also shown promise in combinations of chemotherapy and immunotherapy [167], and polyADP ribose polymerase inhibitors capitalizing on synthetic lethality in certain breast and ovarian cancers [168]. Other more information-based combination strategies such as evolutionary steering [169] and resistance reversal [170] have shown proof of concept.

Combination therapies nevertheless have several notable drawbacks. First, they can fail if not carefully devised. Hegreness *et al.* [171] showed how synergistic antibiotic combinations could accelerate the evolution of resistance compared to (*a priori*, less preferred) antagonistic combinations (see also [96]). Second and similarly, the use of multiple tactics could mean that lower intensities of each are employed to limit toxicity; these lower doses are more likely to fall within the ‘mutant selection window’ should such resistant strains be present, and result in resistance evolution [101, 172]. Third, it is logistically more challenging to administer combinations than monotherapies. This extends both to the willingness of medical practitioners to employ unfamiliar novel therapies and for patients to follow protocols.

## POTENTIAL LIMITATIONS

The disease ecosystem framework focuses on a well-studied host (humans) and a small number of their well-studied BEAs (most examples from bacteria and cancer). The remarkable diversity of host species and BEAs and their intra-population variation—but also the fact that many BEAs are associated with multiple disease ecosystems within the same host individual, or exploit multiple host species, or have complex life cycles with intermediate hosts—mean that the framework presented here is only an initial step towards a more refined general framework and more taxon-specific versions. This situation is no different from the challenges of understanding classic ecosystems, and much of the progress in identifying process and pattern in the latter should be useful in instructing approaches to understanding disease ecosystems.

Second and similarly, many BEA taxa do not replicate in all hosts (particularly for complex life-cycle parasites, such as helminth worms), meaning that *in situ* mutation is not a factor. These species are confronted with some of the same basic constraints as are parasites that can replicate and evolve within their host (resource levels, competition, immune responses), and consequently have evolved mechanisms to plastically adapt to the host ecosystem and in particular, regulate host immunity [173]. The present framework could be extended to accommodate these BEAs.

Third, the ecosystem framework notably ignores host-to-host transmission of BEAs and the spread of resistance between hosts. This concern is evidently not relevant to cancers but is an important factor for infectious diseases, where there is a potential conflict between the immediate interests of individual patients and future interests of the greater population (e.g. [7, 108, 163, 174, 175]).

A fourth limitation is the need for sufficiently rich information to employ certain therapeutic measures, particularly those aiming to contain the BEA. Some BEAs can be quantified either directly or through correlations with measurable signs of disease (e.g. biomarkers, scans). Quantifying ecosystem components such as the immune system (e.g. [176]) could show promise as performance indicators of natural or therapeutically influenced BEA control.

## CONCLUDING REMARKS

The immense body of knowledge on classic ecosystems can contribute to our understanding of disease ecosystems. We will however need more than these analogies to achieve a predictive theory of disease ecosystem dynamics. The principal reason is their underlying complexity. Healthy organism ecosystems and their responses to disease have evolved to maintain homeostasis/performance and remediation to homeostasis, respectively. Although the former should be fairly predictable, the latter will be dynamic, context dependent and more challenging to forecast. The disease ecosystem framework represents an initial step toward understanding disease, predicting its course and designing eco-evolutionarily sensible therapies.

Disease ecosystems are complex and dynamic and have no *a priori* spatial limits. The ecosystem framework focuses on proximal interactions at the host cellular and tissue levels. These interactions will interlock with other tissue and organ systems in the host individual, and ultimately influence and be influenced by population and external environmental-scale systems. Maintaining generality even at the cellular and tissue scales necessarily means omitting considerable realistic detail (see e.g. Table 2 in [177]). Moreover, at aggregate levels, ecological and evolutionary dynamics are likely to be complex given the feedback structures of homeostatic regulation [27], interaction networks [14], and stochasticity in the mutation process and the host’s immune response [178, 179]. Despite this complexity, it is encouraging that across two pervasive BEA types in humans (bacteria and cancer) there is broad similarity in the basic interactions of the disease ecosystem (this study), and with regard to therapies, correspondences in drug resistance evolution [115]. This suggests generality in the framework presented here.

The ecosystem framework is a scaffolding. To be fully operative, it needs to be supplemented with quantitative descriptions of each component process. Although the framework on its own can advise specific types of therapeutic decisions (either ones where resistance is not an issue or satisficing is the only option—i.e. State 1 and late State 3), it will be limited without parameterized models to guide finer containment approaches such as adaptive therapies (States 2 and 3; see e.g. [111]). Such a model would link the most important components of the ecosystem to the variables of interest, typically BEA population size, frequency of resistance, virulence factors or aggressiveness, and disease signs. The model would then be used to evaluate how different candidate therapeutic interventions affect the likelihood that an objective will be achieved. Usually but not always, preventing or limiting BEA evasion (e.g. resistance) will be an integral part of which measure(s) is (are) finally employed.

We are only beginning to scratch the surface of how the disease ecosystem functions and its relevance as part of a larger network, including other ecosystems in the individual, the population and as part of wider (classic) ecosystems [180]. Understanding these processes, their interactions, and importance in creating pattern at different scales will be considerable challenges (Box 4), but promise to lead to novel insights about the ecology and evolution of symbioses, and new breakthroughs in conceptualizing and treating disease.

**Box 4: Questions for future research**

- How do disease ecosystems change with host diet, sex and age?
- To what extent do ecology and evolution in classic ecosystems produce patterns that resemble those in healthy and diseased host ecosystems?
- Does the likelihood of BEA resistance differ between natural (e.g. boosting existing immune responses) and novel (e.g. synthetic drug) interventions?
- How important is spatial heterogeneity (spatial distributions of immune cells, microbiota and BEAs) and indirect interactions (local nutrients mediating competition with microbiota or influencing the immune system) to BEAs and disease dynamics?
- What characteristics of the disease ecosystem predict pathogenicity, within-host migration, and (for parasites) host to host transmission?
- What are the common features and specificities in disease ecosystems found in different tissues and organs, host individuals within a population, closely related species and distantly related taxa?
- The ecosystem framework should be applicable to both healthy and disrupted within-organism systems more generally (e.g. due to injury, aging, non-BEA associated diseases). Is there a useful common framework across organism–ecosystem types?

## GLOSSARY

1. **Disease.** Changes to the structure and function of host cells, tissues or organ systems associated with interactions between the BEA and the host.
2. **Signs.** Objective observations of disease, typically of macroscopic features, such as swollen tonsils consistent with strep throat.
3. **Symptoms.** Subjective assessments of disease, such as pain or discomfort.
4. **Pathogenicity.** The propensity for disease to result in tissue damage, morbidity and mortality. Like disease, pathogenicity is a property of the interaction between the BEA and host.
5. **Resistance**. A BEA phenotype that protects from specific antagonisms such as immune responses or therapies.
6. **Cure**. Disease remediation to no detectable signs and symptoms.
7. **Chemotherapy**. The use of chemicals to treat disease, usually focused at killing the etiological agent, either microparasites or tumor cells.
8. **Eradication**. The complete elimination of the BEA.
9. **Containment**. Maintaining disease below an acceptable maximum threshold.
10. **Microparasites**. Microbial parasites that replicate within their host, including viruses, bacteria, fungi and protozoa.
11. **Virulence factors**. BEA traits that increase BEA fitness (growth, survival and reproduction) and intensification of disease (i.e. pathogenicity).
12. **Inflammation**. Alteration of the disease ecosystem that promotes the intensification of the immune response and remediation of disease damage. Chronic inflammation can result in tissue damage.
13. **Satisficing**. An acceptable improvement in disease signs and symptoms.

## Supplementary Material

Supplementary Data
