# Supplementary material for: An ecosystem framework for understanding and treating disease
Source: Evol Med Public Health. 2018 Oct 9;2018(1):270–86. doi: 10.1093/emph/eoy032 (PMC6252061; doi:10.1093/emph/eoy032)
Supplement: Supplementary Data [file eoy032_supp.docx]

**SUPPLEMENTARY INFORMATION**

**A simple criterion**

The accuracy of predicting chemotherapeutic outcomes will be limited by information about the disease ecosystem. Therapeutic impact on the BEA will depend on its population size, resistance or routes to escape, and interactions with components of the disease ecosystem such as the immune system. Moreover, drugs (or other therapies) may interact with the disease ecosystem in additional ways to their intended objective.

As a first step towards a more informative criterion of therapeutic success, I introduce a constant term for therapeutic escape as a simplification of heterogeneity in the mortality risk (Hochberg & Holt 1999). Assume *n* distinct asexual, haploid classes (e.g., strains) of BEA where for class *i* during the time interval *t* to *t+*Δ the current population size is N_i,t_, maximum growth rate is λ_i_, and density dependent limitation (e.g., through competition and/or predation) is *f*_i,t_. A key parameter is ϕ_i_, which encapsulates various mechanisms leading to overall strain sensitivity to the drug.

Population changes over any time interval *t* to *t+*Δ can be expressed using the standard recursion

N_i,t+Δ_ = λ_i_ ϕ_i_ *f*_i,t_{N_i,t_} N_i,t_.

Assuming that resistant or protected variants are initially rare, and that once chemotherapy begins the BEA population is impacted such that the remaining individuals are not sufficiently numerous to compete for limiting nutrients or space, then after *x* intervals, clone *i* can be approximated by

N_i,t+_*_x_*_Δ_ = (λ_i_ ϕ_i_)*^x^* N_i,0_,

where N_i,0_ is the population of strain *i* after therapy. N_i,0_ for any given strain may be positive either because the strain is resistant, or if the therapy is not applied at sufficient intensities or duration.

As long as the proportion of BEAs not impacted by the therapy is less than the reciprocal of the population growth rate (ϕ_i_ < 1/ λ_i_ for all *i* strains), then the therapy will reduce BEA numbers. More interestingly, the criterion for successfully achieving population control below a target density, T is

W = Σ_i_ (λ_i_ ϕ_i_)*^x^* N_i,0_ < T. (1)

Further, note the following. First, positive growth is always exponential meaning that the ϕ to achieve the control objective can be highly sensitive to the multiplier λ. Second, for criterion (1) to obtain *all* genotypes *i* must be sufficiently controlled. Third, should a single escape mutant result in positive growth, then it takes a minimum of ln(T)/ln(λ_m_) time units for therapeutic failure. And fourth, although not explicitly modelled here, the effects of the immune system could be included in a simple way as reductions in λ; these reductions could accrue through time (expansion of the innate or acquired immune responses), meaning that W could actually increase or Σ_i_ (λ_i_ ϕ_i_)*^x^* >1 before the impact of the immune system reverses this inequality. This also highlights the overly simplistic nature of criterion (1) (akin to the Norton-Simon Hypothesis whereby dose and duration of effective treatment will depend on growth kinetics; see Michor and Beal 2015 for overview applied to cancers) when dynamic processes in the ecosystem (such as immune responses) are accounted for, since W could exceed T for a time, after which increased impact on the BEA would lower growth such that W<T.

Hochberg ME, Holt RD. The uniformity and density of pest exploitation as guides to success in biological control. In: Hawkins BA, Cornell HV (eds). *Theoretical Approaches to Biological Control.* Cambridge: Cambridge University Press, 1999, 71-88.

Michor F, Beal K. Improving Cancer Treatment via Mathematical Modeling: Surmounting the Challenges Is Worth the Effort. *Cell* 2015;**163**:1059–63.
